# Leaf Age-Dependent Effects of Foliar-Sprayed CuZn Nanoparticles on Photosynthetic Efficiency and ROS Generation in *Arabidopsis thaliana*

**DOI:** 10.3390/ma12152498

**Published:** 2019-08-06

**Authors:** Ilektra Sperdouli, Julietta Moustaka, Orestis Antonoglou, Ioannis-Dimosthenis S. Adamakis, Catherine Dendrinou-Samara, Michael Moustakas

**Affiliations:** 1 Department of Botany, Aristotle University of Thessaloniki, GR-54124 Thessaloniki, Greece; 2 Institute of Plant Breeding and Genetic Resources, Hellenic Agricultural Organisation–Demeter, Thermi, GR-57001 Thessaloniki, Greece; 3 Department of Plant and Environmental Sciences, University of Copenhagen, Thorvaldsensvej 40, DK-1871 Frederiksberg C, Denmark; 4 Laboratory of Inorganic Chemistry, Department of Chemistry, Aristotle University of Thessaloniki, 54124 Thessaloniki, Greece; 5 Department of Botany, Faculty of Biology, National and Kapodistrian University of Athens, 157 84 Athens, Greece

**Keywords:** bimetallic nanoparticles, hydrogen peroxide, mature leaves, non-photochemical quenching, photoprotective mechanism, photosynthetic heterogeneity, plastoquinone pool, redox state, spatiotemporal heterogeneity, young leaves

## Abstract

Young and mature leaves of *Arabidopsis thaliana* were exposed by foliar spray to 30 mg L^−1^ of CuZn nanoparticles (NPs). The NPs were synthesized by a microwave-assisted polyol process and characterized by dynamic light scattering (DLS), X-ray diffraction (XRD), and transmission electron microscopy (TEM). CuZn NPs effects in *Arabidopsis* leaves were evaluated by chlorophyll fluorescence imaging analysis that revealed spatiotemporal heterogeneity of the quantum efficiency of PSII photochemistry (Φ*_PSΙΙ_*) and the redox state of the plastoquinone (PQ) pool (*q*_p_), measured 30 min, 90 min, 180 min, and 240 min after spraying. Photosystem II (PSII) function in young leaves was observed to be negatively influenced, especially 30 min after spraying, at which point increased H_2_O_2_ generation was correlated to the lower oxidized state of the PQ pool. Recovery of young leaves photosynthetic efficiency appeared only after 240 min of NPs spray when also the level of ROS accumulation was similar to control leaves. On the contrary, a beneficial effect on PSII function in mature leaves after 30 min of the CuZn NPs spray was observed, with increased Φ*_PSΙΙ_*, an increased electron transport rate (ETR), decreased singlet oxygen (^1^O_2_) formation, and H_2_O_2_ production at the same level of control leaves.An explanation for this differential response is suggested.

## 1. Introduction

Both zinc (Zn) and copper (Cu) are essential elements for plant growth [1]. Zn deficiency results in a rapid inhibition of plant growth and development, while several physiological processes are impaired [1,2,3]. Zinc scarcity in arable soils [4] is a major problem worldwide [2], which is mainly due to the low Zn soil solubility resulting in Zn unavailability to plant roots [5]. Adequate Zn supply is suggested to improve productivity and nutrients in crops [6]. Low Zn concentrations in soils can be improved by adding Zn fertilizers, but this is a costly and ineffective policy [7]. However, to increase grain Zn concentrations, foliar Zn application can be applied [7,8,9]. As Cu is also an essential element for all organisms, Cu-based fertilizers and fungicides have been widely used in agriculture as well [10,11].

Nanoparticles (NPs) in agriculture are used to reduce the amount of sprayed chemical products by the smart delivery of active ingredients, diminish nutrient losses in fertilization, and increase yields through optimized water and nutrient management [12,13,14,15]. Nevertheless, NPs are experiencing problems in reaching the market as novel products in agriculture, making agriculture still a negligible area for nanotechnology due mainly to the high production costs required in high volumes and to doubtful practical profits and lawmaking uncertainties [15]. In addition, the major concern regarding the use of NPs is their possible phytotoxicity, which is closely related to their chemical composition, structure, size, and surface area [12].

Since plant production is driven by photosynthesis, it is possible to estimate the fate of plant growth and development by evaluating photosynthetic function [16]. The process of photosynthesis converts light energy into chemical energy by the collaboration of photosystem I (PSI), and photosystem II (PSII), which work in coordination [16,17,18]. Chlorophyll fluorescence analysis has been widely used as a highly sensitive indicator of photosynthetic efficiency [19,20,21,22,23,24,25,26]. The obtained information can be interpreted to acquire knowledge about the state of the photosynthetic machinery and the effects of environmental pressure on plants [27,28]. Nevertheless, photosynthetic functioning is not uniform at the leaf area particularly under abiotic stress circumstances, which makes conventional chlorophyll fluorescence measurements non-characteristic of the physiological status of the entire leaf [29]. This disadvantage overcomes chlorophyll fluorescence imaging analysis and permits the detection of spatiotemporal heterogeneity at the total leaf surface [30,31,32,33,34,35].

The synthesis of hydrophilic CuZn NPs is challenging and has been hardly reported before [36,37]. We have previously evaluated the phytotoxicity of 15 mg L^−1^ and 30 mg L^−1^ of CuZn NPs sprayed on tomato plants by determining their effects on the light reactions of photosynthesis [38]. The evaluated CuZn NPs displayed minimal ionic dissolution (<10%), a significant amount of biocompatible polyol surface coating (32%), and high crystallinity, factors that minimize their toxic effects [39]. While no significant effects in PSII functionality were noticed with 15 mg L^−1^ of NPs, the application of 30 mg L^−1^ of CuZn NPs resulted in a reduced plastoquinone (PQ) pool that gave rise to H_2_O_2_ generation [38]. A reduced PQ pool reflects an imbalance between energy supply and demand [40,41]—or, in other words, excess excitation energy [42,43,44]. Young leaves have the ability to dissipate the excess excitation energy by non-photochemical quenching (NPQ) more efficiently than mature leaves [45], and this is sufficient in scavenging reactive oxygen species (ROS) [42,46,47]. Based on these reports, we hypothesized that exposing young and mature leaves to 30 mg L^−1^ of CuZn NPs will result in differential effects on them, with young leaves retaining a more oxidized PQ pool and less H_2_O_2_ accumulation. *Arabidopsis thaliana* was selected as the plant material to test our hypothesis, since it has been widely used as a model system in understanding the physiological mechanisms of higher plants [31,48].

## 2. Materials and Methods

### 2.1. Synthesis of CuZn NPs

Based on our previous results, a modified synthesis of CuZn NPs was followed using a commercial microwave accelerated reaction system, the Model MARS 6-240/50-CEM [38]. Equal amounts of Zn(NO_3_)_2_·4H_2_O (2.0 mmol) and Cu(NO_3_)_2_·3H_2_O (2.0 mmol) were mixed and dissolved in 20 mL of triethylene glycol (TrEG). After centrifugation at 2800 g, the supernatant liquids were discarded, and the black-brown precipitate was washed three times with ethanol. NPs were re-dispersed in water via sonication assistance to form stable aqueous suspensions at concentrations <200 mg L^−1^. 

### 2.2. Characterization of CuZn NPs

Primary particle size and morphology was determined by a conventional transmission electron microscope (TEM) (JEOL JEM 1010, Tokyo, Japan) [38]. 

The crystal structure was investigated through X-ray diffraction (XRD) performed on a Philips PW 1820 diffractometer (Amsterdam, The Netherlands) at a scanning rate of 0.050/3 s, in the 2θ range from 10° to 90°, with monochromatized Cu Kα radiation (λ = 1.5406 nm) [38].

The hydrodynamic size of CuZn NPs was determined by dynamic light scattering (DLS) measurements, which were carried out at 25 °C utilizing a Nano ZS Zetasizer (Malvern Instrument, Worcestershire, UK) apparatus [33]. 

### 2.3. Plant Material and Exposure to CuZn NPs

*Arabidopsis thaliana* ecotype Columbia (Col-0) seeds obtained from the Nottingham Arabidopsis Stock Centre (NASC) were sown on a soil and peat mixture in a controlled-environment growth chamber under 22 ± 1/18 ± 1 °C day/night temperature with a 16-hour day at 130 ± 20 μmol photons m^−2^ s^−1^ light intensity and 50 ± 5/60 ± 5% day/night humidity. Four and six-week-old Arabidopsis plants were spayed with 30 mg L^−1^ of CuZn NPs. Rosette leaf 8 from four-week-old (young, immature leaf) and six-week-old (mature to senescing leaf) were selected for photosynthetic measurements 30 min, 90 min, 180 min, and 240 min after the foliar spray with 30 mg L^−1^ of CuZn NPs, while control plants were sprayed with distilled water.

### 2.4. Chlorophyll Fluorescence Imaging Analysis

Chlorophyll fluorescence analysis was conducted with an Imaging-PAM Chlorophyll Fluorometer (Walz, Effeltrich, Germany) as described in detail previously [27]. Chlorophyll fluorescence parameters were measured in dark-adapted (15 min) leaves (rosette leaf 8) from four and six-week-old Arabidopsis plants sprayed with distilled water (control), or 30 mg L^−1^ of CuZn NPs. In each leaf, eight to 11 areas of interest (AOI) that covered the whole leaf area were selected for analysis. Four to five leaves from different plants were measured at each treatment at the actinic light intensity of 140 μmol photons m^−2^ s^−1^. By using the Imaging Win software (Heinz Walz GmbH, Effeltrich, Germany), we measured the effective quantum yield of photochemistry in PSII (Φ*_PSII_*), the quantum yield of regulated non-photochemical energy loss in PSII (Φ*_NPQ_*), the quantum yield of non-regulated energy loss in PSII (Φ*_NO_*), the photochemical quenching (*q*_p_) that is a measure of the redox state of the PQ pool, the non-photochemical quenching (NPQ), and the relative PSII electron transport rate (ETR).

Representative results of the effective quantum yield of photochemical energy conversion in PSII (Φ*_PSΙΙ_*) and the redox state of PQ pool (*q*_p_) are also shown as color-coded images, after 5 min of illumination with 140 μmol photons m^–2^ s^–1^.

### 2.5. Imaging of ROS

The presence of ROS was detected in young and mature leaves of *Arabidopsis thaliana* by staining with 25 µM of 2′,7′-dichlorofluorescein diacetate (H_2_DCF-DA, Sigma, St. Louis, MO, USA) in the dark, as described before [41]. After 30 min of incubation, the leaves were observed with a Zeiss AxioImager Z.2 fluorescence microscope (Jena, Germany) equipped with an MRc5 Axiocam using the AxioVision SE64 4.8.3 software according to the manufacturer’s instructions. 

### 2.6. Statistical Analyses

Four to five leaves from different plants were analyzed for each treatment, and the differences between chlorophyll fluorescence parameters were separated by paired t-test at a level of *p* < 0.05 with the StatView software (computer package, Abacus Concepts, Inc., Berkley, CA, USA) [23]. 

## 3. Results

### 3.1. Characterization of the Synthesized CuZn NPs

**Figure 1:**
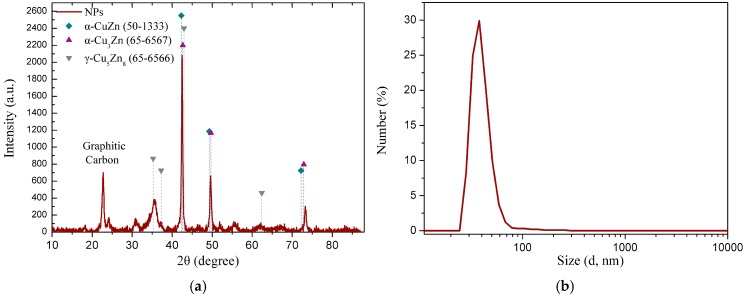
The X-ray diffraction (XRD) patterns of CuZn nanoparticles (NPs) (**a**), and the size distribution (diameter in nm) of the aqueous suspensions of CuZn NPs evaluated by dynamic light scattering (DLS) numbers measurements (**b**).

**Figure 2:**
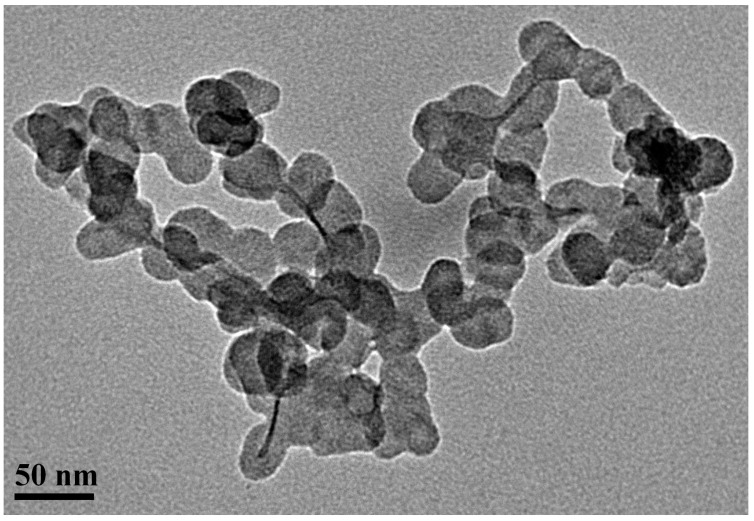
Size and morphology of CuZn NPs determined by transmission electron microscopy.

The crystal structure of the NPs was investigated through X-ray diffraction (XRD) (Figure 1a). The observed peaks at 35.84°, 42.4°, 49.78°, and 73.47° are attributed to γ-brass (JCPDS no. 5-6566) and α-brass (JCPDS no. 50-1333 and no. 65-6567), while no significant changes were observed from the previous reported by us CuZn NPs [38]. The composition analysis of the NPs by inductively coupled plasma (ICP) indicated a 52%/48% copper/zinc proportion, respectively, and thus an overall composition of α-Cu_47_Zn_29_/γ-Cu_9_Zn_15_ based on the X-ray diffractions. TEM images of CuZn NPs (Figure 2) revealed small, spherical nanoparticles in the range of 20 nm to 30 nm in contrast to the formation of nanoclusters [38]. The different nanoarchitecture is attributed to the half amount of polyol (TrEG) that has been used in the present synthesis.

CuZn NPs are hydrophilic, and thus readily disperse in water. The hydrodynamic diameter provided by DLS number measurements (Figure 1b) was 35 nm, matching well to the size provided by TEM and indicated monodispersity. Additionally, the amount of leached ions in a 30 mg L^−1^ aqueous suspension of CuZn NPs after 24 h of incubation was found to be 1.8 mg L^−1^ for Cu and 2.2 mg L^−1^ for Zn, respectively. 

### 3.2. Changes in Lght Energy Partitioning at PSII in Young and Mature Leaves After Exposure to CuZn NPs

**Figure 3:**
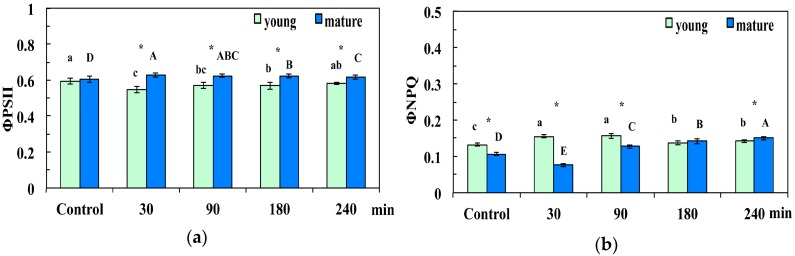
Changes in the quantum efficiency of photosystem II (PSII) photochemistry (Φ*_PSΙΙ_*) (**a**), and the quantum yield of regulated non-photochemical energy loss in PSII (Φ*_NPQ_*) (**b**); of *Arabidopsis thaliana* young and mature leaves measured (at 140 μmol photons m^−^^2^ s^−^^1^) 30 min, 90 min, 180 min, and 240 min after the foliar spay with 30 mg L^−1^ of CuZn NPs or distilled water (control). Error bars on columns are standard deviations based on four to five leaves from different plants. Columns with different letters (lowercase for young leaves and capitals for mature) are statistically different (*p* < 0.05). An asterisk (*) represents a significantly different mean of the same time treatment between young and mature leaves (*p* < 0.05).

We estimated the light energy partitioning at PSII, that is, Φ*_PSII_*, Φ*_NPQ_* and Φ*_NO_*, which sum to one. The quantum yield of photochemical energy conversion (Φ*_PSΙΙ_*) at 30 min, 90 min, and 180 min after the foliar spray with 30 mg L^−1^ of CuZn NPs presented a significant decrease in young leaves, while in mature leaves, it increased significantly compared to controls (Figure 3a). Φ*_PSΙΙ_* recovered to control values in young leaves 240 min after the spray, while at the same time remaining significantly higher than controls in mature leaves (Figure 3a). Φ*_PSΙΙ_* in mature leaves at 30 min, 90 min, 180 min, and 240 min after spraying with CuZn NPs was significantly higher than young leaves (Figure 3a). 

The quantum yield of regulated non-photochemical energy loss (Φ*_NPQ_*) at 30 min, 90 min, 180 min, and 240 min after the CuZn NPs spray increased significantly, compared to control, in young leaves (Figure 3b). Φ*_NPQ_* 30 min after the NPs spray decreased in mature leaves, while it increased significantly afterwards, compared to control (Figure 3b). Φ*_NPQ_* in young control leaves, and at 30 min and 90 min after the CuZn NPs spray was significantly higher than that in mature leaves (Figure 3b). In comparison, Φ*_NPQ_* was significantly higher in mature leaves 240 min after the spray with CuZn NPs (Figure 3b). 

**Figure 4:**
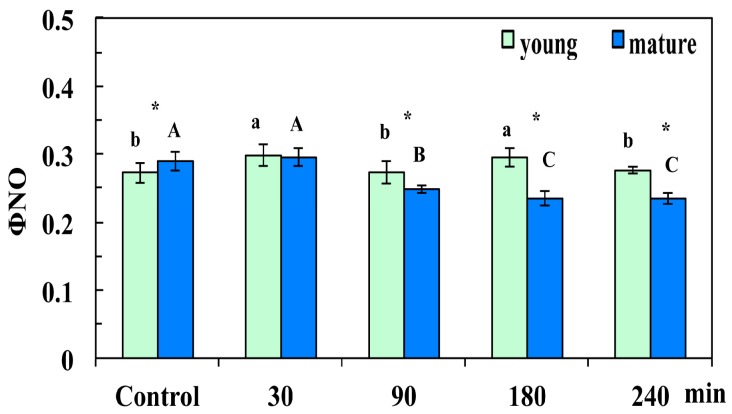
Changes in the quantum yield of non-regulated energy dissipation in PSII (Φ*_NO_*) of *Arabidopsis thaliana* young and mature leaves measured (at 140 μmol photons m^−^^2^ s^−^^1^) 30 min, 90 min, 180 min, and 240 min after the foliar spay with 30 mg L^−1^ of CuZn NPs or distilled water (control). Error bars on columns are standard deviations based on four to five leaves from different plants. Columns with different letter (lower case for young leaves and capitals for mature) are statistically different (*p* < 0.05). An asterisk (*) represents a significantly different mean of the same time treatment between young and mature leaves (*p* < 0.05).

The quantum yield of non-regulated energy loss (Φ*_NO_*), which is a loss process due to PSII inactivity, increased significantly in young leaves 30 min after the CuZn NPs spray compared to control, while it remained unchanged in mature leaves, where it decreased significantly later on (90 min, 180 min, and 240 min after the foliar spray) (Figure 4). In young leaves, Φ*_NO_* 90 min after the foliar spray with NPs decreased to control values, and increased later on (180 min), but retained control values 240 min after spraying with the NPs (Figure 4). Φ*_NO_* in mature control leaves was significantly higher than that in young leaves, but at 90 min, 180 min, and 240 min after the foliar spray with 30 mg L^−1^ of CuZn NPs, it decreased significantly compared to young leaves and control values (Figure 4).

### 3.3. Changes in the Photoprotective Energy Dissipation and the Electron Transport Rate in Young and Mature Leaves After Exposure to CuZn NPs

**Figure 5:**
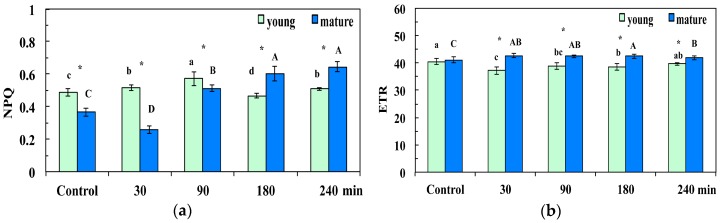
Changes in the non-photochemical fluorescence quenching (NPQ) (**a**), and the relative PSII electron transport rate (ETR) (**b**); of *Arabidopsis thaliana* young and mature leaves measured (at 140 μmol photons m^−^^2^ s^−^^1^) 30 min, 90 min, 180 min, and 240 min after the foliar spay with 30 mg L^−1^ of CuZn NPs or distilled water (control). Error bars on columns are standard deviations based on four to five leaves from different plants. Columns with different letter (lower case for young leaves and capitals for mature) are statistically different (*p* < 0.05). An asterisk (*) represents a significantly different mean of the same time treatment between young and mature leaves (*p* < 0.05).

The non-photochemical quenching (NPQ) increased significantly at 30 min and 90 min after the CuZn NPs spray in young leaves, compared to the control, while it decreased 180 min after spraying, and increased again significantly 240 min after spraying (Figure 5a). NPQ in mature leaves decreased 30 min after spraying with NPs, but later on (90 min, 180 min, and 240 min after the foliar spray), it increased compared to control values (Figure 5a). NPQ was significantly higher in young leaves compared to mature and control leaves and 30 min and 90 min after the CuZn NPs spray, but significantly lower than in mature leaves at 180 min and 240 min after the foliar spray (Figure 5a).

The relative electron transport rate at PSII (ETR) decreased significantly in young leaves 30 min, 90 min, and 180 min after the foliar spray with 30 mg L^−1^ of CuZn NPs, while at the same time it increased significantly in mature leaves compared to controls (Figure 5b). ETR recovered to control values in young leaves 240 min after the spray, while it remained significantly higher than controls in mature leaves (Figure 5b). The ETR in mature leaves at 30 min, 90 min, 180 min, and 240 min after the spray with CuZn NPs was significantly higher than that in young leaves (Figure 5b).

### 3.4. Changes in the Redox State of Plastoquinone (PQ) Pool in Young and Mature Leaves After Exposure to CuZn NPs

**Figure 6:**
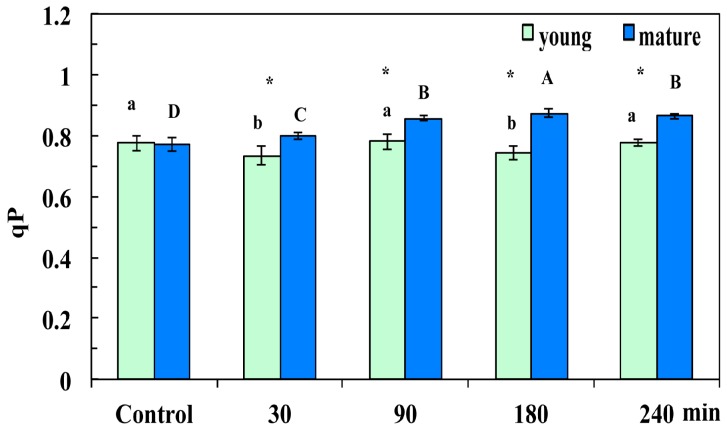
Changes in the photochemical fluorescence quenching, which is the relative reduction state of the plastoquinone (PQ) pool, reflecting the fraction of open PSII reaction centers (*q*_p_) of young and mature *Arabidopsis thaliana* leaves measured (at 140 μmol photons m^−2^ s^–1^) 30 min, 90 min, 180 min, and 240 min after the foliar spay with 30 mg L^−1^ of CuZn NPs or distilled water (control). Error bars on columns are standard deviations based on four to five leaves from different plants. Columns with different letter (lower case for young leaves and capitals for mature) are statistically different (*p* < 0.05). An asterisk (*) represents a significantly different mean of the same time treatment between young and mature leaves (*p* < 0.05).

The redox state of PQ pool (*q*_p_), which is a measure of the fraction of open PSII reaction centers, decreased significantly at 30 min and 180 min after the CuZn NPs spray in young leaves, compared to control; in contrast, it was at control values 90 min and 240 min after spraying (Figure 6).

At all the sampling periods (30 min, 90 min, 180 min, and 240 min after the NPs spray), the mature leaves were in a more oxidized state than control (Figure 6).

### 3.5. Spatiotemporal Heterogeneity of the Quantum Efficiency of PSII Photochemistry and the Redox State of Plastoquinone (PQ) Pool in Young and Mature Leaves After Exposure to CuZn NPs

**Figure 7:**
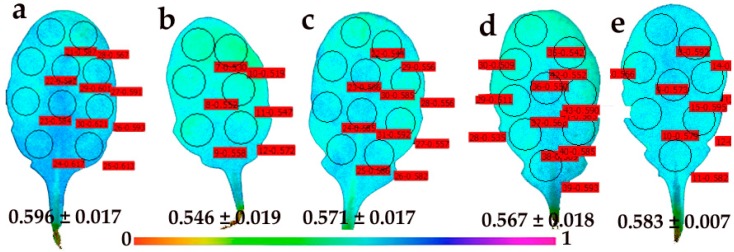
Representative chlorophyll fluorescence images of the effective quantum yield of PSII photochemistry (Φ*_PSΙΙ_*) of *Arabidopsis thaliana* young leaves after 5 min of illumination at 140 μmol photons m^−^^2^ s^–1^. Leaves were measured after the foliar spray with distilled water (control) (**a**), or 30 min (**b**), 90 min (**c**), 180 min (**d**) and 240 min (**e**) after the foliar spay with 30 mg L^−1^ of CuZn NPs. The color code depicted at the bottom of the images ranges from values 0.0 to 1.0. The areas of interest (AOI) are shown in each image. The average Φ*_PSΙΙ_* value of all the AOI for the whole leaf is shown.

The quantum yield of photochemical energy conversion (Φ*_PSΙΙ_*) in control young leaves showed a spatial heterogeneity, with higher values in the midrib of the leaves than in the lamina (Figure 7a). A spatiotemporal heterogeneity of Φ*_PSΙΙ_* in young leaves was evident 30 min after the foliar spray with 30 mg L^−1^ of CuZn NPs with lower values in the distal (tip) leaf area (Figure 7b). The spatiotemporal heterogeneity of Φ*_PSΙΙ_* in young leaves 90 min after the foliar spray was still evident due to an increase of whole leaf Φ*_PSΙΙ_* values (Figure 7c), and became amplified 180 min after spraying (Figure 7d). Then, 240 min after spraying with CuZn NPs, Φ*_PSΙΙ_* increased to the control whole leaf values, showing also a spatial heterogeneity, with higher Φ*_PSΙΙ_* values in the area where lower values were previously scored (distal leaf area) (Figure 7e).

**Figure 8:**
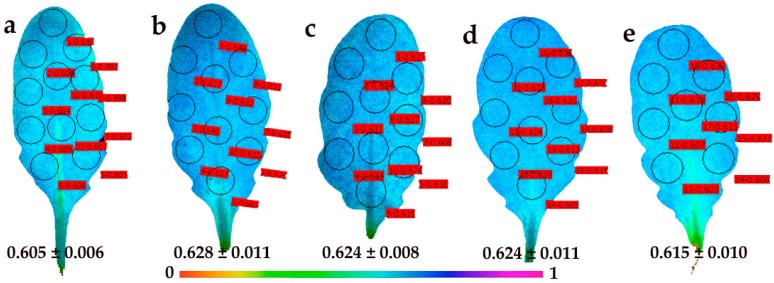
Representative chlorophyll fluorescence images of the effective quantum yield of PSII photochemistry (Φ*_PSΙΙ_*) of *Arabidopsis thaliana* mature leaves after 5 min of illumination at 140 μmol photons m^−^^2^ s^–1^. Leaves were measured after the foliar spray with distilled water (control) (**a**), or 30 min (**b**), 90 min (**c**), 180 min (**d**) and 240 min (**e**) after the foliar spay with 30 mg L^−1^ of CuZn NPs. The color code depicted at the bottom of the images ranges from values 0.0 to 1.0. The areas of interest (AOI) are shown in each image. The average Φ*_PSΙΙ_* value of all the AOI for the whole leaf is shown.

Mature control leaves (Figure 8a) presented less spatial heterogeneity in Φ*_PSΙΙ_* compared to control young leaves (Figure 7a), with higher values in the distal (tip) leaf area (Figure 8a). Higher values also occurred in the same area 30 min after the foliar spray with 30 mg L^−1^ CuZn NPs, which also caused the whole leaf Φ*_PSΙΙ_* to increase (Figure 8b). The spatiotemporal heterogeneity of Φ*_PSΙΙ_* in mature leaves was also evident 90 min after spraying with NPs (Figure 8c), but become less apparent 180 min after the foliar spray (Figure 8d). At 240 min after the foliar spray, Φ*_PSΙΙ_* decreased in mature leaves in the whole leaf area, but remained higher than in the control leaves (Figure 8e).

**Figure 9:**
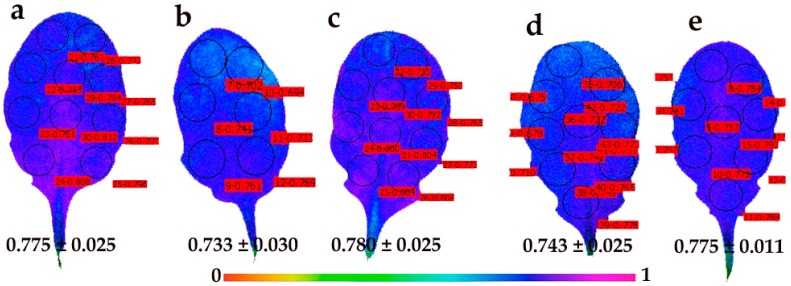
Representative chlorophyll fluorescence images of the relative reduction state of the plastoquinone (PQ) pool, that is, the photochemical fluorescence quenching, reflecting the fraction of open PSII reaction centers (*q*_p_), of *Arabidopsis thaliana* young leaves after 5 min of illumination at 140 μmol photons m^−2^ s^–1^. Leaves were measured after the foliar spray with distilled water (control) (**a**), or 30 min (**b**), 90 min (**c**), 180 min (**d**), and 240 min (**e**) after the foliar spay with 30 mg L^−1^ of CuZn NPs. The color code depicted at the bottom of the images ranges from values 0.0 to 1.0. The areas of interest (AOI) are shown in each image. The average *q*_p_ value of all the AOI for the whole leaf is shown.

Images of the redox state of the PQ pool (*q*_P_) of control young leaves showed a spatial heterogeneity, with higher values in the proximal (base) midrib of leaves (Figure 9a), as observed in the images of Φ*_PSΙΙ_* (Figure 7a). At 30 min after the foliar spray with 30 mg L^−1^ of CuZn NPs, a spatiotemporal heterogeneity of *q*_P_ in young leaves was noticed, with lower values in the distal (tip) leaf area (Figure 9b) and significantly lower whole leaf *q*_P_ values than those of the young control leaves (Figure 9a). At 90 min after the foliar spray with CuZn NPs, the *q*_P_ images of young leaves (Figure 9c) were similar to the images of control young leaves (Figure 9a). At 180 min after the foliar spray with CuZn NPs, the whole leaf *q*_P_ values in young leaves decreased (Figure 9d), resembling the images 90 min after the foliar spray (Figure 9c) with less evident heterogeneity. At 240 min after spraying with NPs, whole leaf *q*_P_ values (Figure 9e) resemble those of control young leaves (Figure 9a).

**Figure 10:**
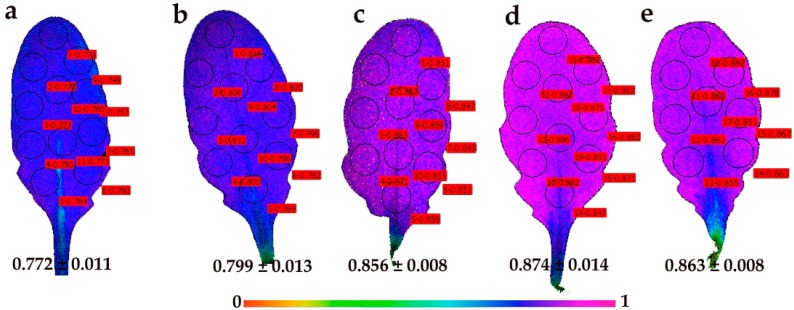
Representative chlorophyll fluorescence images of the relative reduction state of the plastoquinone (PQ) pool—that is, the photochemical fluorescence quenching, reflecting the fraction of open PSII reaction centers (*q*_p_) of *Arabidopsis thaliana* mature leaves after 5 min of illumination at 140 μmol photons m^−2^ s^–1^. Leaves were measured after the foliar spray with distilled water (control) (**a**), or 30 min (**b**), 90 min (**c**), 180 min (**d**) and 240 min (**e**) after the foliar spay with 30 mg L^−1^ of CuZn NPs. The color code depicted at the bottom of the images ranges from values 0.0 to 1.0. The areas of interest (AOI) are shown in each image. The average *q*_p_ value of all the AOI for the whole leaf is shown.

Images of the redox state of the PQ pool (*q*_P_) of control mature leaves showed leaf homogeneity rather than leaf heterogeneity (Figure 10a). At 30 min after the foliar spray with 30 mg L^−1^ of CuZn NPs, a slight heterogeneity of *q*_P_ was observed in mature leaves, with increased *q*_P_ values in the whole leaf area (Figure 10b). Later on (90 min, 180 min, and 240 min after spraying with CuZn NPs), a further increase of *q*_P_ values compared to the control mature leaves was observed in the whole leaf area (Figure 10c–e).

### 3.6. ROS Generation in Young and Mature Leaves After Exposure to CuZn NPs

**Figure 11:**
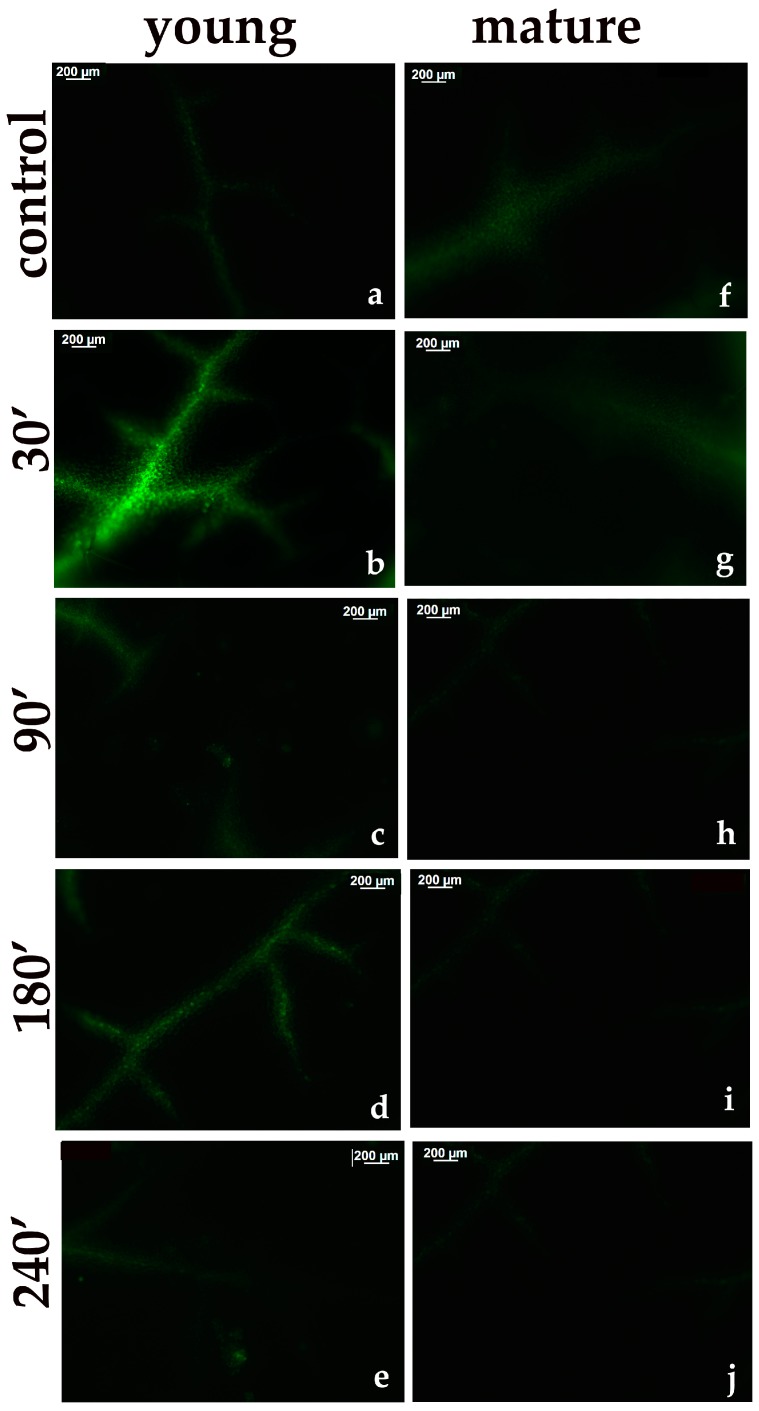
Representative patterns of reactive oxygen species (ROS) (H_2_O_2_) production in *Arabidopsis thaliana* young (**a**–**e**) and mature (**f**–**j**) leaves, as indicated by the fluorescence of H_2_DCF-DA. The H_2_O_2_ generation after the foliar spray with distilled water (control) in a young leaf (**a**) and mature leaf (**f**); or 30 min after foliar spay with 30 mg L^−1^ of CuZn NPs in a young leaf (**b**) and mature leaf (**g**); 90 min after foliar spay with 30 mg L^−1^ of CuZn NPs in a young leaf (**c**) and mature leaf (**h**); 180 min after foliar spay with 30 mg L^−1^ of CuZn NPs in a young leaf (**d**) and mature leaf (**i**); and 240 min after foliar spay with 30 mg L^−1^ of CuZn NPs in a young leaf (**e**), and mature leaf (**j**). Scale bare: 200 µm. A higher H_2_O_2_ content is indicated by the light green color.

ROS generation was quantified in young (Figure 11a–e) and mature (Figure 11f–j) *A. thaliana* leaves by the fluorescent probe DCF-DA. In both young (Figure 11a) and mature (Figure 11f) control leaves, no notable quantities of H_2_O_2_ could be observed. At 30 min after the foliar spray with CuZn NPs, the highest H_2_O_2_ generation was noticed in young leaves (Figure 11b), accompanying the lower measured *q*_P_ values (Figure 9b). At the same time in mature leaves (Figure 11g), the level of ROS accumulation was similar to the control values (Figure 11f). At 90 min after the CuZn NPs spray, almost no H_2_O_2_ could be detected in young leaves (Figure 11c). In mature leaves, no H_2_O_2_ could be detected at 90 min, 180 min, and 240 min after the CuZn NPs spray, either (Figure 11h–j). At 180 min after spraying with CuZn NPs, a high H_2_O_2_ production (but substantially less than 30 min after spraying) was observed in young leaves (Figure 11d). At 240 min after spraying with CuZn NPs, the level of ROS accumulation in young leaves (Figure 11e) was similar to that of the control (Figure 11a).

## 4. Discussion

Inorganic NPs are emerging as novel agrochemicals due to their unique characteristics and high surface energy, which make them effective in lower doses compared to conventional inorganic ionic formulations. For instance, bulk brass has been utilized in the healthcare industry, while ionic forms of zinc and copper such as Bordeaux mixture, sulfate, and chloride salts are used in agrochemistry, but with adverse environmental effects and toxicity. However, due to the low water solubility, these ionic forms of agrochemicals are applied in relatively large amounts in order to effectively control the phytopathogens when the spores vegetate, which is through causing the secretion of malic acid and amino acids and subsequently dissolving them [49]. As a consequence, the limit between plant protection and phytotoxicity is still a matter of discussion. A need exists for new products that are going to have high biological activity and less metal in the formulation. Under these perspectives, hydrophilic CuZn NPs retain the desired characteristics of bulk brass, while forming stable aqueous suspensions with minimal ionic dissolution that are effective in low doses.

Young leaves can utilize only a fraction of absorbed irradiance in photochemical reactions via CO_2_ assimilation, since light capture ability develops earlier than CO_2_ assimilation capacity [45,50,51]. When the absorbed light is not used in photochemistry, in order to avoid photodamage, the excess excitation energy has to be safely removed by a photoprotective mechanism called non-photochemical quenching (NPQ) [52,53]. Consequently, the ability to dissipate excess excitation energy by NPQ is higher in young leaves than in mature leaves [45,47], which means that under control growth conditions, NPQ is significantly higher in young leaves compared to mature leaves (Figure 5a). 

Heterogeneity in PSII photochemistry has been frequently reported to depend on the leaf age [41,42,43,45,47,48]. We observed changes in light energy partitioning related to leaf age under control growth conditions mostly related to Φ*_NPQ_* and Φ*_NO_*. Control young leaves had higher Φ*_NPQ_* than mature leaves (Figure 3b), and without any significant difference in Φ*_PSII_* (Figure 3a), it resulted in significantly lower Φ*_NO_* (Figure 4). However, 90 min, 180 min, and 240 min after the NPs spray, Φ*_NO_* increased in young leaves compared to mature leaves (Figure 4) due to a decreased photochemical energy conversion (Φ*_PSΙΙ_*) (Figure 3a) that could not be compensated by the increased Φ*_NPQ_* (Figure 3b). Φ*_NO_* consists of chlorophyll fluorescence internal conversions and intersystem crossing, which leads to the formation of singlet oxygen (^1^O_2_) via the triplet state of chlorophyll (^3^chl*) [41,54,55,56], thus suggesting increased ^1^O_2_ formation in young leaves compared to mature leaves. NPQ is one of the most important photoprotective mechanisms in plants [25,41,57,58]. The enhancement of NPQ that reflects the dissipation of excess excitation energy in the form of harmless heat in young leaves (Figure 5a) at 30 min after spraying with CuZn NPs, could not protect young leaves from ROS generation at 30 min after the NPs spray (Figure 11b). However, the increase of NPQ in young leaves 90 min after the CuZn NPs spray (Figure 5a) was effective at retaining the same redox state of the PQ pool with control leaves and reducing H_2_O_2_ production at 90 min after the NPs spray to control levels (Figure 11c). An effective photoprotection can be attained only if NPQ is adjusted in such a way that no changes occur in the redox state of the PQ pool [41,59]. Otherwise, an imbalance between energy supply and demand occurs, indicating excess excitation energy [57,58,59]. Under such circumstances, the generation of H_2_O_2_ occurs (Figure 11b), which can be diffused through the leaf veins to act as a long-distance signaling molecule [38,41,60,61,62]. The intracellular ROS signaling pathways are initiated by the redox state of the PQ pool that regulates photosynthetic gene expression, comprising also a mechanism of plant acclimation [38,63,64]. The redox state of the PQ pool is of unique significance for antioxidant defense and signaling [65]. It has been shown recently that ROS generation is influenced also by the circadian system [66,67]. We postulate that ROS generation at 30 min after the NPs spray (Figure 11b) possibly served as the signaling molecule to contribute to a more oxidized state of the PQ pool at 90 min after the NPs spray (Figure 6, Figure 9c), resulting in a H_2_O_2_ production similar to the control leaf level (Figure 11c).

The foliar spay of *Arabidopsis thaliana* young and mature leaves with 30 mg L^−1^ of CuZn NPs revealed a spatiotemporal heterogeneity of Φ*_PSΙΙ_* and *q*_p_ measured (at 140 μmol photons m^−2^ s^–1^) 30 min, 90 min, 180 min, and 240 min after spraying (Figure 7, Figure 8, Figure 9 and Figure 10). Young leaves show a higher spatial heterogeneity (Figure 7 and Figure 9) compared to mature leaves (Figure 8 and Figure 10). Nevertheless, PSII function was not uniform for both leaf types, making conventional chlorophyll fluorescence instruments not suitable for abiotic stress studies and pointing out the advantages of using chlorophyll fluorescence imaging analysis in the recognition of spatial heterogeneity at the leaf surface [29,30,31,32,33,34,35]. The response of cells to the same stress condition is not uniform, with some cells behaving more vulnerably than others [68].

In contrast to previous reports that young leaves acclimatize better to environmental changes and can maintain a better ROS homeostasis [43,45,69], mature leaves responded better. Thus, in disagreement with our hypothesis, the PSII photochemistry of young leaves seem to be negatively influenced when exposed to 30 mg L^−1^ of CuZn NPs. Young leaves could overcome the negative effects on the function of PSII only after 240 min of the NPs spray, at which point the level of ROS accumulation was also similar to that of control young leaves. On the contrary, a beneficial effect was observed in the PSII function of mature leaves 30 min after the CuZn NPs spray, which was through an increased quantum efficiency of PSII photochemistry (Φ*_PSΙΙ_*), an increased electron transport rate (ETR), an increased fraction of open PSII reaction centers (*q*_p_), decreased ^1^O_2_ formation, and no notable changes in H_2_O_2_ generation.

Zinc and Cu are important micronutrients that are required for plant growth and development [1,38], but when they are in excess, they can cause toxicity effects on plant growth and development, affecting photosynthetic function [3,70]. In young leaves with sufficient Zn and Cu concentrations, the spray with 30 mg L^−1^ of CuZn NPs resulted in an excess supply of them, causing negative effects on PSII function and increased ROS production. Increased H_2_O_2_ generation in young leaves after 30 min of spraying with 30 mg L^−1^ of CuZn NPs (Figure 11b) was correlated to a lower oxidized state of the PQ pool (Figure 9b). Zinc is involved in a wide variety of physiological processes, playing catalytic, regulatory, and structural roles with several crucial functions in the cell [1,71,72,73], but excess Zn has to be detoxified in roots by sequestration to protect the sensitive photosynthetic leaf tissues [3,72]. Zn phytotoxicity varies extensively, depending on combinations with other heavy metals, the environmental conditions, the plant species, and the plant age [72], as well as by the leaf age, as shown here. 

Leaf senescence turns leaves from units with a primary assimilation role into centers of nutrient mobilization [74,75]. During leaf senescence, new metabolic pathways are activated and others are de-activated, with nutrient and material remobilization, followed by a declining photosynthesis [74,75]. The *A. thaliana* rosette leaf 8 from six-week-old plants is a mature to senescing leaf; thus, nutrient remobilization occurs, resulting in nutrient deficiency. Spraying these leaves with 30 mg L^−1^ of CuZn NPs restores Zn and Cu deficiency and improves photosynthetic efficiency. Zinc is known to contribute to the repair processes of PSII by turning over the photodamaged D1 protein [72,76]. Copper is vital for photosynthesis, and more than half of Cu is found in chloroplasts participating in the light reactions [77]. The foliar spraying of Cu NPs induced stress tolerance by stimulating antioxidant mechanisms [78]. However, this nutrient remobilization explanation has yet to be established.

Although plants are producers and play a key role in the ecosystem, the impact of NPs upon them is not well studied [79]. In order to understand the uptake, transport, and also bioaccumulation of NPs in plants after foliar exposure, different qualitative and quantitative methods are still being developed with an unclear comparability of results among the different techniques [80,81]. Among the different techniques, inductively coupled plasma mass spectroscopy (ICP-MS) is one of the most reliable methods for the detection of NPs, offering a range of advantages in high detection limits and high sensitivity for many elements [79,81,82,83].

Previously, both the positive and harmful impacts of NPs on terrestrial and aquatic plants have been established, which are mainly due to the concentration, size, and specific surface area of NPs, the exposure methodology, and the plant species that was examined [38,79,81,84,85,86]. In the root uptake of NPs, translocation to the above-ground parts takes place in a unidirectional pathway through xylem vessels, while in the foliar uptake of NPs, translocation takes place in bidirectional pathways throughout the plant by the phloem [85]. The efficiency of uptake and translocation, and the effects of NPs on growth metabolism and photosynthesis vary from plant to plant [84]. Some studies report that the foliar application of NPs considerably increases the chlorophyll content in plants and results in a higher amount of light energy capture and photosynthesis enhancement [84], while another study reports that the root uptake of NPs decreases Φ*_PSΙΙ_* and *q*_p_, and increases Φ*_NO_* due to an ineffective photoprotective mechanism (NPQ) resulting from a significant decrease of the PsbS protein, which is the key regulator of the energy dissipation process [87]. Nevertheless, the investigation on NPs is still at an initial phase; more laborious work is required in order to understand their impact on the physiological, biochemical, and molecular mechanisms in plants [79,84].

The present study demonstrates that considering the leaf developmental stage is important for understanding the mechanisms underlying leaf growth responses to environmental stresses [41,42,43,88]; thus, it must be taken into account in environmental stress studies in order to compare leaves of the same developmental stage [41,42,43,48,89]. Leaves of distinct ages differentially control stress responses, and plant responses against biotic and abiotic stresses are balanced in a leaf age-dependent manner [90].
